# Transactions of the American Gynæcological Society

**Published:** 1877-10

**Authors:** 


*Transactions of the American Gynæcological Society. Volume I. For the Year* 1876. Boston: H. O. Houghton & Co. 1877. Buffalo: Peter Paul & Bro.

The American Gynæcological Society was organized on the third of June 1876, with an original fellowship of thirty-nine. The first annual meeting of the Society was held on the 13th, 14th and 15th of Sept. 1876, in New York City and the volume of Transactions now before us is the result of the meeting. The annual address by the President of the Society, Dr. Fordyce Barker of New York, is a thoughtful and concise statement of the objects to which the Society should devote itself and the mode in which its proceedings should be conducted. From a hasty review of the history of obstetric and gynæcic literature during the last century he makes a favorable and hopeful prediction of the advances which should be made in the coming one. With increased and rapid means of intercommunication science becomes cosmopolitan, and he predicts that soon in Obstetrics those who call themselves the followers of any one school as the English, the French or the German will be, as they are rapidly becoming, unknown.

The next article in order is by Dr. Thomas Addis Emmet upon the Etiology of Uterine Flexures, with the Proper mode of Treatment Indicated. Dr. Emmet's operation is familiar to all of our readers and most of them are doubtless familiar with the paper by Dr. Peaslee in which he took issue with Dr. Emmet upon this very subject. Dr. Emmet's division of the cervix uteri for the relief of dysmenorrhœa dependent upon flexions is made with scissors. In the discussion which followed there was somewhat of a tendency we think to be a little conserative and refrain from extensive division of the cervix. We can imagine and and readily call to mind cases in which Dr. Emmet's long and deep cut would have a tendency to shorten the cervix, open up the cervical canal and accomplish in this manner the desired end, but there are however, many we think a majority of cases, in which a less radical operation would be of equal value. The paper is full of carefully collected statistics and valuable facts and deserves a careful and considerate study. We cannot at this time give all the papers such notice as we would desire; we can only notice some of them by title reserving any particular notice for the more important ones. Following Dr. Emmet's is a paper by Dr. Skene of Brooklyn upon cicatraces of the vagina and cervix uteri. Dr. Battey of Georgia follows with an article upon Extirpation of the functionally active Ovaries for the remedy of otherwise incurable disease. Dr. Battey proposes in the paper an operatian so radical that it deserves if for no other reason some notice. His operation has been before the public for some time under the name of Normal Ovariotomy. An unfortunate name we think, for neither is the operation normal nor is it for the removal of the normal ovary if the ovary removed is the cause of the "otherwise incurable disease."

Dr. Battey has made ten operations of this kind with the following results: —Two have died. Case one both ovaries removed, decrease of pain and other symptoms to total disappearance increased strength and weight. Has uterine hemorrhages appearing at irregular intervals and lasting from two to five weeks, sometimes exhausting. Case two, one ovary removed, pain relieved but subsequently appeared in right ovary which enlarged. Case three both removed; success; no appearance of hemorrhage. Case four. One ovary removed, relief of pain so great that she wishes other extirpated. Case five. One removed pain appeared in other ovary which was removed. Case six. Both ovaries removed. Died. Case seven, Both ovaries removed, result perfect. Case eight, Both removed result unsatisfactory. Case nine, same as case five second ovary removed but with unsatisfactory result. Case ten. Both removed. Died. In all but the first case the operation was made through the vaginal cul-de- sac. The discussion upon Dr. Battey's paper was postponed until the meeting in 1877.

Following were papers by Dr. J. Matthews Duncan upon Central rupture of the perniæum; by Dr. E. W. Jenks upon Vibrum Prunifolium in the treatment of Diseases of Women; by Dr. Parvin, An Illustration of Xenomenia; by Dr. Robert Barnes on the Relations of Pregnancy to General Pathology, an exceedingly interesting and suggestive paper. A paper by Dr. Wm. H. Byford upon the Spontaneous and Artificial Destruction and Expulsion of Fibrous Tumors of the Uteras.

We find at this point that we are exceeding our space and hence shall have to omit all mention of the other valuable papers presented which were twelve in number together with a graceful and carefully prepared Memoir of Gustav Simon, prepared by Dr. Munde. The memoir is accompanied by a finely executed steel plate of the eminent surgeon.

The volume is an example of what the Transactions of Medical Societies should be, and we predict that if it continues as it has commenced that the Gynæcological Society will be the model which other and older organizations will be compelled to follow. B.

